# The “Irradiance Effect” Plays a Crucial Role in the Photosensitization of *Escherichia coli* by Blue Light

**DOI:** 10.3390/molecules30234515

**Published:** 2025-11-22

**Authors:** Fabrizio Bolognese, Nataliia Emashova, Valerio Baldelli, Paolo Landini, Viviana Teresa Orlandi

**Affiliations:** 1 Department of Biotechnology and Life Sciences (DBSV), University of Insubria, Via J.H. Dunant 3, 21100 Varese, Italy; fabrizio.bolognese@uninsubria.it (F.B.); nemashova@uninsubria.it (N.E.); 2 Department of Biosciences, University of Milan, 20133 Milan, Italy; valerio.baldelli@unimi.it (V.B.); paolo.landini@unimi.it (P.L.)

**Keywords:** antimicrobial Blue Light, aBL, photooxidation

## Abstract

Antimicrobial Blue Light (aBL) can be used to control the growth of pathogens in several applicative fields, from sanitization of inert surfaces to human skin treatment and from industry to food. Though the mechanism of action is still unknown, it has been hypothesized that specific wavelengths can activate potential endogenous photosensitizers in microbial cytoplasm and/or envelope. In turn, this photooxidative stress could induce inactivation of macromolecules resulting in bacterial killing. In this work, we investigated the effect of radiometric parameters of light at 410 nm on *Escherichia coli* K-12 MG1655, a strain rather tolerant to blue light irradiation. Interestingly, by changing the radiometric parameters of aBL protocol, different rates of killing were observed. Irradiation at 100 J/cm^2^ caused a variable antimicrobial effect depending on the irradiance values. We observed an “irradiance effect”: namely, at higher irradiance values, the inhibitory effect is reduced. On the other hand, at increasing fluences the bactericidal rate increases. In addition, the shift from continuous to pulsed light could enhance the antimicrobial activity of protocols using higher irradiance values. Taken together, these results underline the importance of defining radiometric parameters to ensure the efficacy of aBL treatments and emphasize the importance of further research into the aBL mechanism.

## 1. Introduction

As far back as 5000 BC, ancient Egyptians used sunlight to disinfect and heal chronic wounds and ulcers [1]. At the beginning of the last century, ultraviolet (UV) radiations generated from quartz and mercury vapor lamps were used to treat acne, psoriasis, syphilis, leprosy, and pellagra. However, increasing data on UV carcinogenic propensity, and its potential to cause skin wrinkles and dermatological diseases, encouraged investigations on the potential use of visible light against bacteria, fungi, and viruses [2,3]. In particular, the antimicrobial effects of blue light, attributed especially to the wavelength range between 400 and 450 nm, gave rise to the “aBL” (antimicrobial Blue Light) technique. In addition, several reports demonstrated the antimicrobial efficacy of longer wavelengths of 460, 465 and 470 nm, respectively [4]. Antimicrobial Blue Light is a “drug free” approach that differs from the well-known photodynamic technique that needs the administration of exogenous photosensitizers (PSs). The irradiation of PSs with visible light matching with their corresponding absorbance peaks elicits the mechanism I and/or mechanism II pathways responsible for the transfer of energy and/or electrons, respectively, from PSs to oxygen. The consequent formation of Reactive Oxygen Species (ROS) and/or singlet oxygen (^1^O_2_) damages cellular components and compromises bacterial viability [5,6]. Even if the mechanism of action of aBL is still under investigation, it has been hypothesized that blue light could excite endogenous photosensitizing chromophores (i.e., iron-free porphyrins and/or flavins) in microbial cells, triggering the production of cytotoxic ROS [7,8]. Given that putative endogenous photosensitizers, such as protoporphyrin, coproporphyrin, and uroporphyrin, are intermediate species in the heme biosynthesis, it is likely that they are accumulated in the cytoplasmic environment [9,10]. In addition, porphyrin-based cytochromes belonging to electron transport chains in the cytoplasmic membrane could act as endogenous PSs [11]. The specific contribution of both extracellular molecules and environmental components that could be activated by blue light should also be taken into proper account. As a consequence, ROS could induce oxidative damage to cytoplasmic membrane macromolecules, lipids, cell wall-associated proteins, and nucleic acids, but also compromise polysaccharides and eDNA of biofilm extracellular matrix [12].

In nature, phototrophic bacteria are able to counteract photooxidative stress arising from photosynthetic reactions through an arsenal of accessory pigments such as primary and secondary carotenoids [13]. Among the non-phototrophic bacteria, under blue light irradiation at high fluences, *Escherichia coli* shows a photophobic response characterized by increasing tumble rates and leading to cell paralysis and death [14]. Thus, this response could be targeted to reduce *E. coli* persistence as an etiological agent of acute and chronic infections or as a fecal contaminant in wastewaters and food/feed samples.

Several reports describe the antimicrobial effect of blue light on *E. coli*. MacLean observed that a fluence of light at 410 nm equal to 68 J/cm^2^ caused a decrease of 3.1 log units of *E. coli* samples collected at high bacterial concentration (10^9^ cfu/mL) [15]. In the report of Halstead, light at 405 nm showed a certain degree of activity against two different strains of *E. coli* [16]. More recently, dos Anjos reported that light at 410 nm was successful in killing *E. coli* strains independently of antibiotic resistance and virulence profiles [17]. In this regard, the increasing spread of multidrug-resistant strains highlights the urgent need to develop alternative antimicrobial strategies to combine with new or old antibiotics and sanitization approaches.

A systematic review and meta-analysis by Lawrence et al. [18] reported that, despite the extensive literature on blue light and *E. coli*, a serious shortcoming in most studies is the absence of clear documentation on the effect of biological and environmental parameters, suggesting improvements for study protocols for future investigations. In this study, the authors tried to fill the gap, testing the activity of light at 410 nm on different strains of *E. coli* to evaluate any interspecific differences. Furthermore, the effect of radiometric set-up (irradiance, fluence rate, mode of irradiation) was considered.

## 2. Results

### 2.1. Effect of Irradiance in Photoinactivation of Escherichia coli

In this study, we examined the response of the model microorganism *Escherichia coli* K-12 MG1655 to different aBL protocols based on LED emission at 410 nm. Irradiation at wavelengths between 395 and 425 nm was already reported to be the most efficient in killing this microorganism [18,19], one that is particularly tolerant among Gram-negative bacteria [15,20,21]. Also, in our previous experiments, irradiation at 410 nm was more efficient than that at 455 nm in killing the *Pseudomonas aeruginosa* PAO1 strain [22]. The light emission from the LED device was modulated by changing irradiance (energy delivered in time unit, in this study expressed as mW/cm^2^) and fluence, respectively. This strategy was aimed at investigating how the delivery of irradiation could, to some extent, increase or modulate the sensitivity of *E. coli* to blue light.

**Figure 1:**
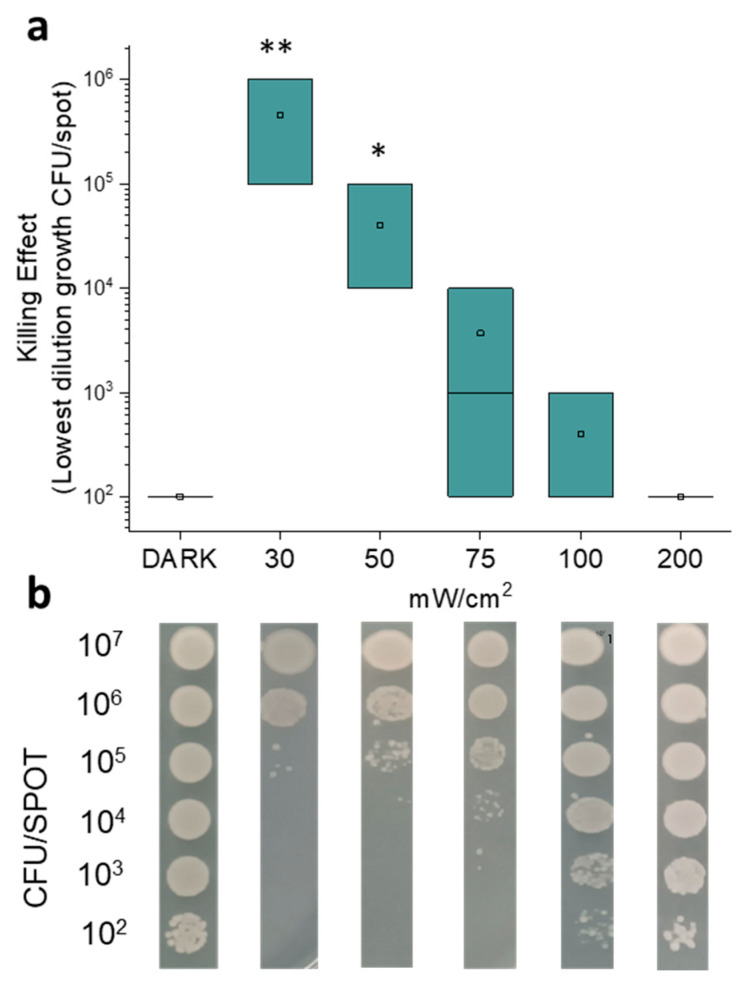
Results of photo-spot test on *E. coli* MG1655 irradiated under light at 410 nm. Bacterial samples were inoculated in LB agar in order to reach the tenfold decreasing densities (from 10^7^ to 10^2^ CFU/spot) and irradiated under increasing irradiance values (30, 50, 75, 100 and 200 mW/cm^2^) to reach 100 J/cm^2^. After 24 h of incubation at 37 °C, the growth of spots was observed and compared to the dark control. In panel (**a**), the graph shows the killing effect expressed as lowest dilution growth values (CFU/spot). The square is the mean value of at least three independent experiments; the green box represents the interquartile range (25–75%). * *p* < 0.05, ** *p* < 0.001 by one-way ANOVA test. In panel (**b**), the representative images of spot growths are depicted.

In particular, the photo-spot test approach was aimed at evaluating the effect of light on the growth of small bacterial samples (volume ~5 μL) inoculated on LB agar at decreasing concentrations (from ~10^7^ to ~10^2^ CFU/spot). Fluence was set at 100 J/cm^2^ and the effect of increasing irradiance values, from 30 to 200 mW/cm^2^, was evaluated. After irradiation and 24 h incubation, as described in the Material and Methods, the growth of single spots was checked. The summarized data of at least three independent replicates (Figure 1a) and the representative images of the photo-spot test (Figure 1b) show that *E. coli* MG1655 growth was inhibited in a manner that is inversely proportional to the irradiance values.

At irradiance values of 30 and 50 mW/cm^2^, we observed the highest antimicrobial effects, which were statistically significant when compared to the dark incubated controls. These irradiation values were efficient in inhibiting the growth of samples up to ~10^5^ and ~10^4^ CFU/spot, respectively. At increasing irradiance values, we observed a gradual decrease in the inhibitory activity of blue light, which was no longer statistically significant. Notably, at 200 mW/cm^2^, the growth was comparable to that of the unirradiated samples. Taken together, these results highlight that the killing effect is inversely proportional to both the irradiance value and the cellular concentration. In the literature, the correlation between irradiance (mW/cm^2^) and the killing effect is still unclear. Huang et al. [19] reported that the efficacy of blue light was proportional to the irradiance value. Matsumoto et al. [23] observed an increase in the killing rate of *E. coli* passing from 0.03 to 0.12 mW/cm^2^, and values from 20 to 40 mW/cm^2^ led to greater antimicrobial activity. Lawrence [18] showed a significant decrease in cell survival (CFU) upon blue light treatment between 5.52 and 50 mW/cm^2^, but values between 60 and 1262.6 mW/cm^2^ did not show such effect. He suggested that, when large irradiance values are used, the number of photons could exceed the concentration of endogenous photosensitizers with a consequent energy loss. Actually, we observed a stronger antimicrobial activity at irradiance values between 30 and 50 mW/cm^2^ with respect to 100 and 200 mW/cm^2^. Blue light irradiation could be compared to other well-known stressors such as heat shock, oxidative stress, antibiotic exposure, and pH variation; furthermore, different responses were reported in bacteria against short-term or long-term stress. Acute damage sensors activate transcriptional regulons by specific sigma factors, while global stress response (GSR) needs long-term integrated adaptation [24]. Long-lasting irradiation could be compared to chronic stress and it seems that *E. coli* is more able to cope with sudden environmental changes than with prolonged exposure to unfavorable conditions. Most importantly, the putative activation of endogenous PSs [18] during aBL might trigger a multi-target photo oxidative stress that is hardly able to be counteracted by random adaptative strategies (i.e., phase or antigenic variation, gene noise, epigenetics). In this scenario, the selection of more fitted bacteria as a result of mutagenic events would be unlikely [25,26].

### 2.2. Effect of Fluence on Photoinactivation of Escherichia coli

**Figure 2:**
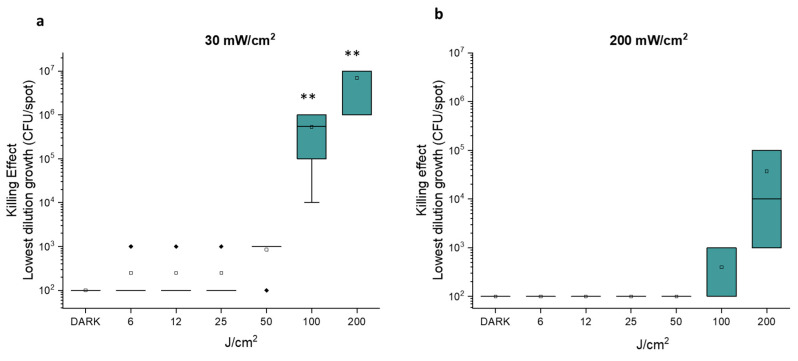
Photo-spot test on *E. coli* MG1655 with light at 410 nm at 30 mW/cm^2^ (**a**) and 200 mW/cm^2^ (**b**). The killing effect reported on the y axis represents the Log_10_ of the lowest cell concentration at which growth is still visible upon treatment with increasing fluence rates, from 6 to 200 J/cm^2^. The square is the mean value of at least three independent experiments, the green box represents the interquartile range (25–75%), the black rhombus represents outlier, and ** *p* < 0.001 by one-way ANOVA test.

The effect of the second radiometric parameter, the fluence (J/cm^2^), was evaluated by settling the irradiance at 30 mW/cm^2^ which proved to be the most antimicrobial in the previous assays, and at 200 mW/cm^2^, which showed no activity. In the photo-spot tests, fluence was increased from 6 to 200 J/cm^2^ (Figure 2).

As shown in Figure 2a, at 30 mW/cm^2^, fluences of 6, 12, 25 and 50 J/cm^2^ did not exert any significant antimicrobial effect. On the other hand, irradiation at 100 J/cm^2^ inhibited the growth of samples at ~10^2^, 10^3^ and 10^4^ CFU/spot. Irradiation at 200 J/cm^2^ inhibited the growth of samples at cell densities up to ~10^6^ CFU/spot. As a result, the last aBL protocol preventing the growth of *E. coli* samples with higher concentrations is the most efficient among those tested. As expected, with an irradiance value of 200 mW/cm^2^, fluences ≤ 100 J/cm^2^ did not induce any antimicrobial effect (Figure 2b). The mild photoinactivation rates observed at 100 and 200 J/cm^2^ were not statistically significant.

We can conclude that, if the relationship between irradiance value and survival rate is still debated [27,28], a general agreement is present on the effect of fluence increase. For example, Huang assessed how the fluences of blue light for inactivation of 90% and 99% of *E. coli* were 81 and 188 J/cm^2^, respectively [19]. 

### 2.3. Comparison of Irradiation Effects at 30 and 200 mW/cm^2^ on E. coli

**Figure 3:**
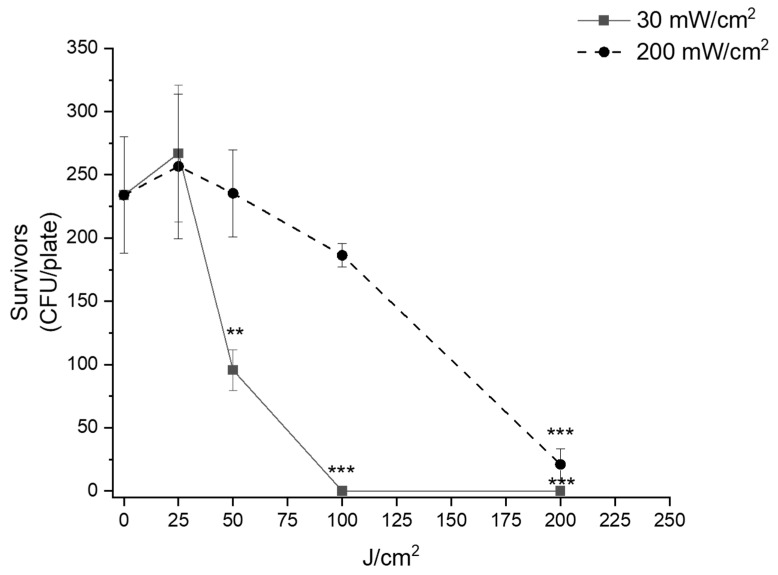
“Irradiance effect” of blue light on *E. coli* MG1655. Bacteria were inoculated on LB agar and dark incubated or irradiated at 30 mW/cm^2^ (continuous line) or 200 mW/cm^2^ (dashed line) at increasing fluence rates. After 24 h incubation at 37 °C, the survivors were evaluated as CFU/plate and represented as media ± standard deviation. The experiments were performed at least three times. ** *p* < 0.001, *** *p* < 0.0001 by one-way ANOVA test.

To deeply investigate the “irradiance effect”, a further assay was used. We inoculated cells on agar plates and evaluated the effects of light on survivors expressed as colony number (CFU/plate). As represented in Figure 3, the “irradiance effect” was clear. The light fluence of 50 J/cm^2^ was significantly (*p* = 0.00114) antibacterial, only if delivered under an irradiance value of 30 mW/cm^2^. Even if at 100 J/cm^2^, both irradiances were efficient in inhibiting the growth of *E. coli* MG1655; the killing rate observed at 30 mW/cm^2^ was ~100% (*p* < 0.0001) and that observed at 200 mW/cm^2^ was ~23% (*p* = n.s.). The highest fluence (200 J/cm^2^) was active both at 30 mW/cm^2^ (*p* < 0.0001) and 200 mW/cm^2^ (*p* < 0.0001).

**Figure 4:**
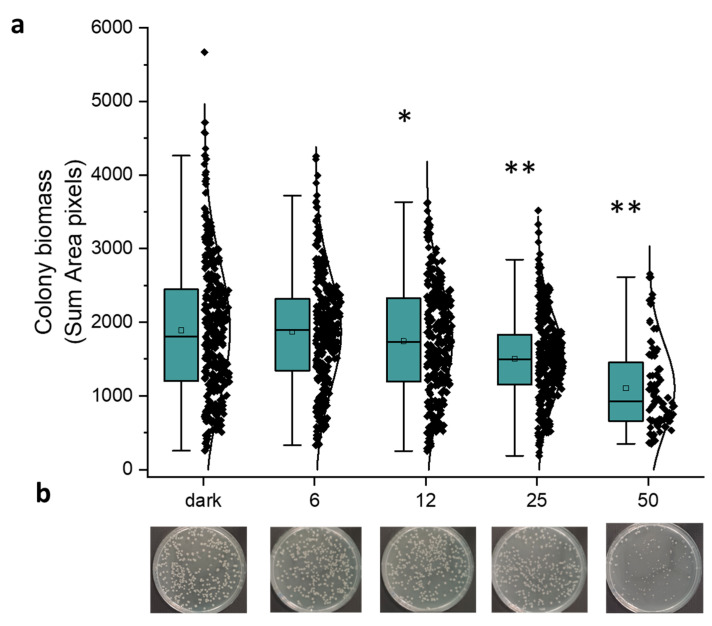
Effect of light at 410 nm on *E. coli* MG1655. Bacteria were inoculated on LB agar and dark incubated or irradiated (30 mW/cm^2^) at increasing fluences (from 6 to 50 J/cm^2^). (**a**) The graph represents the distribution of colony biomass acquired by OpenCFU and expressed as sum area pixels. The green box represents the interquartile range (25–75%), the black rhombus represents outlier, * *p* < 0.001, ** *p* < 0.0001 by one-way ANOVA test. (**b**) Images of the corresponding samples used for the image analysis by OpenCFU.

As described above, the number of colonies (CFU/plate) significantly decreased at fluences equal to or higher than 50 J/cm^2^. We evaluated the antimicrobial effect of blue light at 30 mW/cm^2^ at fluences ≤ 50 J/cm^2^, measuring the area of the single colonies by means of OpenCFU 3.9.0 software. In Figure 4, the graphical distribution of colony biomass, represented as “sum area pixels” at increasing fluences (Figure 4a), refers to one representative experiment (Figure 4b). It is noteworthy that under irradiation at 12 and 25 J/cm^2^ the decrease in the colony dimension is significantly lower (*p* = 0.0312 and *p* < 0.0001, respectively) than in dark incubated controls. Furthermore, the fluence of 50 J/cm^2^ not only prevented the microbial growth at 30 mW/cm^2^ as previously described, but also significantly compromised (*p* < 0.0001) the colony dimensions, as shown in Figure 4b. The same statistically significant trend was observed in another two independent experiments.

**Figure 5:**
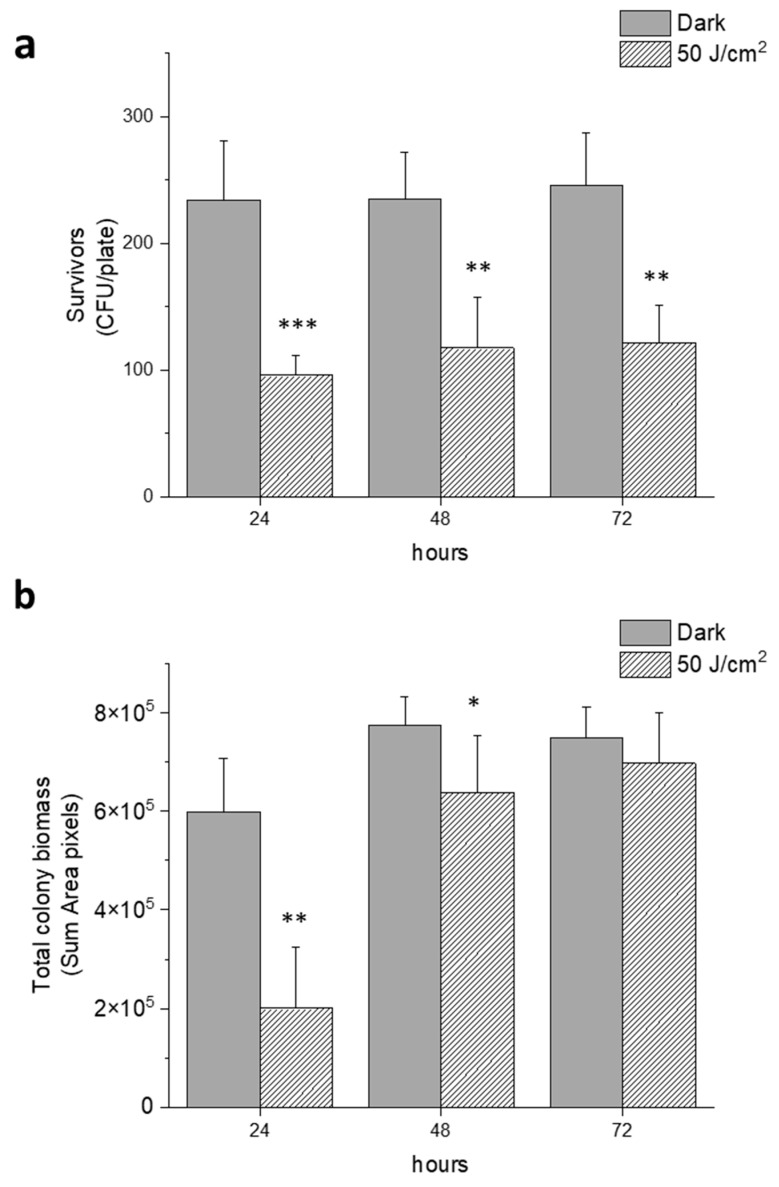
Effect of blue light irradiation on *E. coli* MG1655 at prolonged incubation times. Cells were kept in the dark or irradiated at 410 nm (30 mW/cm^2^ and 50 J/cm^2^) and incubated at 37 °C for 24, 48 and 72 h, respectively. (**a**) Representation of survivors expressed as CFU/plate at increasing incubation times; (**b**) total biomass of bacterial colonies expressed as a sum of area pixels acquired by OpenCFU at increasing incubation times. The experiments were performed at least three times (media ± sd), * *p* < 0.05, ** *p* < 0.001, *** *p* < 0.0001 by one-way ANOVA test.

We further evaluated if the antimicrobial effect of blue light at 50 J/cm^2^, observed after 24 h incubation at 37 °C, was maintained for longer periods. To this aim, the surviving rates (CFU/plate) and the total biomass of all colonies were evaluated 24, 48 and 72 h after treatment. The number of colonies (CFU/plate) formed by survivors on each single plate after irradiation did not change from 24 to 72 h of incubation, and the observed differences were statistically significant (Figure 5a). On the other hand, even if the total biomass of the colonies formed by survivors was significantly smaller than the dark controls, after 72 h of incubation the difference was not statistically significant (Figure 5b). Thus, even if the effect of irradiation was bactericidal (~50%), some physiological impairment could also be hypothesized in surviving cells, as shown by reduced colony size with respect to untreated samples.

In our set-up, both irradiance values (30 and 200 mW/cm^2^) caused a killing effect that was dependent on fluences (J/cm^2^). The delivery of increasing amounts of photons causes an increase in bacterial depletion in samples with the same cellular concentration. Even if irradiation at 30 mW/cm^2^ did not impair cell viability, it caused a significant decrease in the colony dimension (Figure 4). At 50 J/cm^2^, cell viability was compromised ~50% up to 72 h after treatment and the growth of the survivors was delayed. These results suggest that aBL could act as an antimicrobial, even at low fluences, and could be combined with other antimicrobial strategies. Lawrence hypothesized that bacterial endogenous photosensitizers are potential targets of blue light irradiation: at high cellular concentrations, increasing amounts of photons could be necessary to activate PSs [18]. Accordingly, in photo-spot test experiments, we observed that the same light fluence was more efficient in killing samples at the lowest cell density (CFU/spot).

### 2.4. Effect of Pulsed Light on Photoinactivation of E. coli

**Figure 6:**
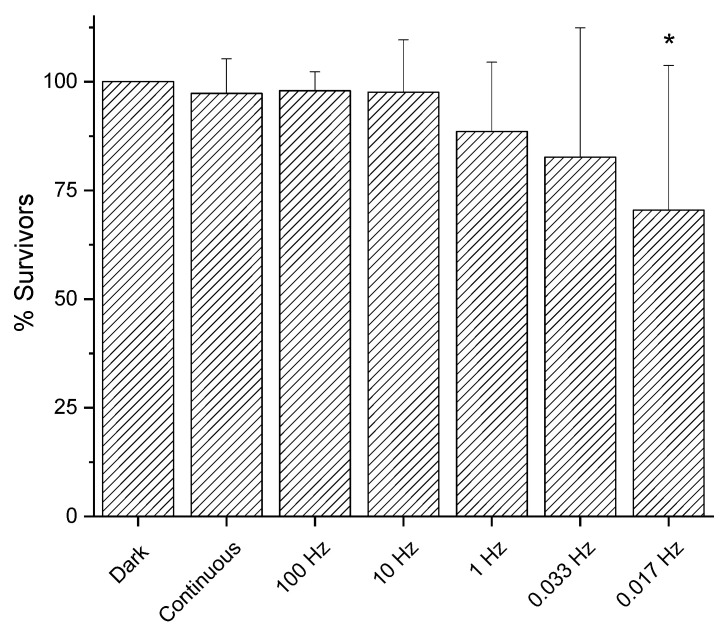
Effect of irradiation mode of light at 410 nm on *E. coli* MG1655. Bacteria were inoculated on LB agar and dark incubated or irradiated at 200 mW/cm^2^ (50 J/cm^2^) with continuous or pulsed light (duty cycle 50%). After 24 h incubation at 37 °C, the survivors were expressed as CFU/plate and used to calculate the percentage of survivors compared to the corresponding dark controls. The y axis represents the percentage of survivors, while the dark incubated control represents 100%. The experiments were performed at least eight times (media ± sd); * *p* < 0.05 by ANOVA test.

The continuous mode of irradiation of light at 410 nm at 200 mW/cm^2^ did not show any effect, even at high fluences (see Figure 2). Thus, we evaluated if the transition from continuous to pulsed light could change the outcome. We tested a pulsed light irradiation set-up with a 50% duty cycle at 200 mW/cm^2^ to reach a fluence of 50 J/cm^2^ under high frequency (from 10 to 100 Hz), and no surviving rate decrease was observed compared to continuous irradiation and dark controls. Interestingly, reaching lower frequency values, a mild antimicrobial effect was detected and, at the lowest frequency value tested, 0.017 Hz, the decrease was statistically significant (*p* = 0.0144) (Figure 6).

Recently, some studies showed that the frequency of pulsed light is a crucial parameter that deserves attention in aBL applications. For example, Huang et al. [29] reported that the viability of *E. coli* ATCC 8739 upon irradiation at 648 J/cm^2^ was influenced by the mode of administration. He obtained a 2.90 log-unit decrease at 4000 Hz, a 2.82 log-unit decrease at 400 Hz, and a 2.44 log-unit for continuous light, respectively. According to Zhang et al. [30], the effect of pulsed frequency on inactivation of *E. coli* MG1655 could be influenced by the kind of duty cycle, as they did not observe statistical differences for frequency values of 10, 100 and 1000 Hz with a constant 50% DC. They also observed that a reduction in duty cycle and light activation time resulted in a considerable improvement in irradiation efficiency: this could be in agreement with our results obtained for 10 and 100 Hz, respectively. The reason was attributed to the process of ROS generation, which consists of two parts: the primary photochemical reaction of the endogenous photosensitizer molecules stimulated by photons, and the secondary response of the conversion of triplet-state oxygen to ROS by excited photosensitizers. With pulsed exposure, the primary and the secondary reactions were stimulated in the turned-on time of a pulsed cycle, while in the turned-off time, excited endogenous photosensitizers likely reset fully before being reactivated again.

### 2.5. Irradiance Effect in Different E. coli Strains

To evaluate if the “irradiance effect” observed for *E. coli* MG1655 was strain-dependent, we considered the two recA1 deficient strains JM109 and DH5α (recombination- and endonuclease-deficient variants). The photo-spot tests were performed, delivering a fluence of 200 J/cm^2^. The results are expressed as the highest inhibition dilution growth values (media ± standard deviation) and summarized in Table 1. In all three bacterial strains the difference observed between irradiation at 30 mW/cm^2^ and 200 mW/cm^2^ was statistically significant. Interestingly, at 200 mW/cm^2^, the recombinase-deficient strains were more sensitive than the wild-type strain. Further investigations are needed to verify if the recombinatory machinery is responsible to some extent for the tolerance to the photooxidative stress of the wild-type strain MG1655. Future studies would also be very intriguing since *E. coli* senses blue light via YcgF protein which carries a BLUF (blue light using Fad) domain that, through cyclic-di-GMP, is involved in biofilm formation [31]. In this scenario, it would be important to evaluate if the detection of blue light emission could trigger cell responses which could ultimately lead to irradiation tolerance.

## 3. Materials and Methods

### 3.1. Bacterial Strains and Growth Conditions

*Escherichia coli* K12 bacterial strains MG1655 (F^−^ lambda- *ilvG*- *rfb*-50 *rph*-1), JM109 (endA1, recA1, gyrA96, thi, hsdR17 (rk^−^, mk^+^), relA1, supE44, Δ(lac-proAB), [F’ traD36, proAB, laqIqZΔM15]) and DH5α (F^−^ φ80*lac*ZΔM15 Δ(*lac*ZYA-*arg*F)U169 *rec*A1 *end*A1 *hsd*R17 (rk^−^, mk^+^) *pho*A *sup*E44 λ^−^*thi*-1 *gyr*A96 *rel*A1) were maintained as glycerol stocks at −80 °C. Cells were inoculated in 100 mL flasks filled with 20 mL of lysogeny broth (LB) and grown overnight at 37 °C with mild agitation. Independent experiments of viable counts for overnight cultures gave average values of 10^9^ cfu/mL.

### 3.2. LED Set-Up

Irradiation of samples was performed with LULab light source (University of Padua, Padua, Italy) [32] that allowed a uniform irradiation of a square area of (75 × 75) mm^2^ with a head composed of 25 high-power LEDs. Each LED was equipped with a specific optic to obtain the maximum collimation of the light on the target. The distance between the LED and the samples was equal to 6 cm. The drive and control systems consisted of a constant current driver and a PWM generator (RIGOL DG1022, Beijing, China) which provided driving signals to control the duty cycle (1–100%) and pulse frequency (1–100 Hz) of the current output. In this experimental work, a 50% duty cycle was used for pulsed light irradiation at 1, 10, 30 and 100 Hz frequency.

### 3.3. Photo-Spot Test

LB overnight cultures of *Escherichia coli* strains were tenfold serially diluted in 96-well plates from 10^9^ to 10^4^ cfu/mL in a final volume of 200 microliters of LB medium. Volumes of 5 microliters were spotted on LB agar medium with a replica device. Single plates were irradiated at 100 J/cm^2^ with irradiance values of 30, 50, 75, 100, and 200 mW/cm^2^, respectively; unirradiated samples were always included as controls. After 24 h of incubation at 37 °C, results were recorded as the presence or absence of spots corresponding to single dilutions. The experiments were performed at least in triplicate.

#### 3.3.1. Fixed Fluence Rate and Increasing Irradiance Values

Experiments on irradiance effects on the growth of test strains were performed at 30, 50, 75, 100 and 200 mW/cm^2^, respectively, to reach a constant fluence of 100 J/cm^2^. Microorganisms were irradiated at 410 nm at room temperature for suitable time intervals and incubated at 37 °C for 24 h. We considered the spot with the lowest bacterial concentration and the antimicrobial effect was expressed as the corresponding lowest dilution growth (CFU/spot).

#### 3.3.2. Fixed Irradiance Values and Increasing Fluence Values

The evaluation of dose–response effects of increasing fluences were based on irradiation at fixed irradiance values (30 mW/cm^2^ or 200 mW/cm^2^, respectively) for increasing times. Selected fluences were 6, 12, 25, 50, 100 and 200 J/cm^2^, respectively. The antimicrobial effect was expressed as the lowest dilution growth (CFU/spot).

#### 3.3.3. Effect of Bacterial Strain

The possible effect of genetic mutations on tolerance/sensitivity against blue light irradiation was evaluated by comparing *Escherichia coli* K12 strains MG1655, JM109 and DH5α, respectively. Irradiation at 30 and 200 mW/cm^2^ was performed with a final fluence of 200 J/cm^2^. Since after 24 h of incubation the irradiation caused the killing of the highest cellular concentration samples, we considered the spot with the highest bacterial concentration at which no growth was visible and the antimicrobial effect was expressed as growth at the highest cell dilution (CFU/spot).

### 3.4. Spread Test

This experimental technique was based on the same rationale of the photo-spot test assay, except that single cells were irradiated instead of bacterial spots. LB overnight cultures of interest were serially diluted in fresh LB medium up to 10^3^ cfu/mL. Volumes of 100 microliters were plated in order to have, on average, 100 to 200 single colonies per plate. After suitable irradiation intervals, samples were incubated at 37 °C as usual. The antimicrobial effect was expressed as CFU/plate. Experiments were performed in triplicate with unirradiated controls. The experiments were performed both under continuous light (30 and 200 mWatt/cm^2^) and pulsed light. Experiments based on pulsed light irradiation were always performed keeping a constant irradiation peak value. Different set-ups were maintained at 200 mW/cm^2^ to reach 50 J/cm^2^. In this experimental work, a 50% duty cycle (DC) was used for pulsed light irradiation at 1, 10, 30 and 100 Hz frequencies. To obtain lower frequency values, 0.033 and 0.017 Hz, alternate irradiation intervals for 15 and 30 s, respectively, were obtained by manual covering or exposing samples to LED light.

### 3.5. Opencfu Processing

Opencfu is an open-source tool that allows quick and accurate image processing with a functional user interface and an optimized software library. Very large sets of images can be processed from capture devices such as webcams. In this experimental work, images were captured at a distance of 22 cm from the plate surface with a 2X magnification. After threshold setting, single components are assessed by a particle filter that scores variables such as area, perimeter, and radius and determines whether or not a region is likely to be valid. Individual objects are accepted and multiple objects are segmented through a variant of watershed algorithm and reassessed by the particle filter. In this experimental work, 90 mm LB agar plates were analyzed with a bilateral threshold of 12 and a minimal radius value of 15. These values allowed reliable detection of colonies in previous control experiments and almost complete correspondence to values obtained from manual colony counting. In addition, Opencfu was used to acquire the biomass dimension of colonies that were expressed as sum area pixel (from 0 to 5000). In addition, the total biomass of all colonies grown on LB agar upon dark incubation and irradiation was measured to evaluate the effect of irradiation for prolonged incubation times.

### 3.6. Statistical Analysis

Statistical analyses of the experimental data (at least three independent tests) were performed using one-way ANOVA and considering data with a *p*-value less than 0.05 as significant.

## 4. Conclusions

This study highlights the pivotal role of the radiometric parameters in aBL applications. The time used by photons to reach *E. coli* cells seems very important: the same fluence exerts a stronger antimicrobial effect when delivered over longer exposure times compared to shorter ones. Even if light at 410 nm at low fluences does not compromise *E. coli* viability, it contributes to the statistically significant microbial growth delay. The transition from continuous to pulsed light at a very low frequency value also produces a certain killing effect. Overall, all these data support the great potential of aBL in many applicative fields, especially when microbial contamination control is needed. In medical care, aBL could be targeted to localized infections such as wound and skin infections, otitis and pneumonia. Promising results could be obtained in the safety of food processing, from disinfection of food surfaces and packaging to environmental decontamination. In addition, agronomic and intensive farming fields could take advantage of this technique.

## Figures and Tables

**Table 1:** Results of photo-spot tests expressed as the highest inhibition dilution growth values (media ± sd) of at least three experiments.

*E. coli* Strain	30 mw/cm^2^	200 mw/cm^2^	*p*
MG1655	7.00 × 10^6^ ± 4.65 × 10^6^	3.67 × 10^4^ ± 5.50 × 10^4^	0.0405
JM109	5.50 × 10^6^ ± 4.81 × 10^6^	3.03 × 10^5^ ± 4.32 × 10^5^	0.0088
DH5α	8.88 × 10^6^ ± 3.18 × 10^6^	1.00 × 10^6^ ± 0.00	*p* < 0.0001

## Data Availability

The data presented in this study are available upon request from the corresponding author.

## Abbreviations

The following abbreviations are used in this manuscript:

aBL	Antimicrobial Blue Light
ANOVA	Analysis of Variance
CFU	Colony-Forming Unit
cm^2^	Centimeter square
DC	Duty Cycle
E	Energy
h	Hour
Hz	Hertz
J	Joule
LB	Lysogeny Broth
LED	Light-Emitting Diode
min	Minute
mW	Milli Watt
PS	Photosensitizer
UV	Ultraviolet
